# Processing Pathways in Mental Arithmetic—Evidence from Probabilistic Fiber Tracking

**DOI:** 10.1371/journal.pone.0055455

**Published:** 2013-01-30

**Authors:** Elise Klein, Korbinian Moeller, Volkmar Glauche, Cornelius Weiller, Klaus Willmes

**Affiliations:** 1 Section Neuropsychology, Department of Neurology, University Hospital, RWTH Aachen University, Aachen, Germany; 2 Interdisciplinary Centre for Clinical Research–Neurofunctional Imaging Lab, University Hospital RWTH Aachen, Aachen, Germany; 3 Knowledge Media Research Center, IWM-KMRC, Tuebingen, Germany; 4 Department of Neurology, Freiburg Brain Imaging, University Medical Center Freiburg, Freiburg, Germany; Centre Hospitalier Universitaire Vaudois Lausanne - CHUV, UNIL, Switzerland

## Abstract

Numerical cognition is a case of multi-modular and distributed cerebral processing. So far neither the anatomo-functional connections between the cortex areas involved nor their integration into established frameworks such as the differentiation between dorsal and ventral processing streams have been specified. The current study addressed this issue combining a re-analysis of previously published fMRI data with probabilistic fiber tracking data from an independent sample. We aimed at differentiating neural correlates and connectivity for relatively easy and more difficult addition problems in healthy adults and their association with either rather verbally mediated fact retrieval or magnitude manipulations, respectively. The present data suggest that magnitude- and fact retrieval-related processing seem to be subserved by two largely separate networks, both of them comprising dorsal and ventral connections. Importantly, these networks not only differ in localization of activation but also in the connections between the cortical areas involved. However, it has to be noted that even though seemingly distinct anatomically, these networks operate as a functionally integrated circuit for mental calculation as revealed by a parametric analysis of brain activation.

## Introduction

Numerical cognition is an integral part of living in complex modern societies. Recent data even suggest that poor numeracy is more detrimental to life perspectives than poor literacy [1]. As a consequence, there is increasing interest in uncovering the neural basis of numerical cognition. However, even though there are numerous fMRI studies localizing the neural correlates of number processing (e.g., see [2] and [3] for reviews; see [4] for a meta-analysis), these studies mainly focused on identifying critical grey matter regions, whereas white matter tracts connecting the respective regions have largely been neglected so far. This is particularly interesting, because the most influential model in numerical cognition, the Triple Code Model (TCM) by Dehaene and colleagues [5]–[8] posits that numerical cognition is subserved by a fronto-parietal network, in which distinct cortical regions need to be connected. Therefore, the current study aimed at investigating white matter connections within this fronto-parietal network of number processing for the first time, employing a probabilistic fiber tracking approach.

### The Triple Code Model of numerical cognition

The TCM of Dehaene and colleagues and its elaborations [5]–[8] is currently the most influential model in numerical cognition because of its unique integration of behavioral and neuro-functional aspects, making it an anatomo-functional model. The model successfully incorporates evidence from studies on brain-lesioned patients, human functional neuroimaging, primate neurophysiology, and developmental neuropsychology in indicating that numerical cognition is subserved by a fronto-parietal network [9]. Generally, the TCM suggests that different aspects of numerical information (e.g., magnitude, facts, parity) are processed in different codes within distinct cerebral regions of the human brain. As a consequence, the TCM proposes three different and task-specific representational codes for the processing of numerical information: First, a bi-hemispheric numerical magnitude representation is supposed, coding numerical quantity [10]–[12]. This magnitude code is assumed to be analogue and is often characterized by the metaphor of a mental number line recruited systematically for the mental manipulation of numerical quantities (e.g. magnitude comparison). This representation is assumed to be subserved by brain areas in the bilateral intraparietal sulcus (IPS), which are suggested to be connected through transcallosal fibers [13]–[15]. Additionally, the bilateral posterior superior parietal lobe (PSPL) seems to support magnitude processing via mental orientation of attention upon the mental number line [8], [16], [17]. Second, a verbal representation of numbers is proposed to be activated in linguistically mediated operations like number naming and counting. Additionally, the arithmetic facts (e.g., multiplication tables) are represented verbally in long term memory, allowing such problems to be solved by arithmetic fact retrieval (e.g., [18]–[21]). Verbal numerical representations are associated with left perisylvian language areas and the left angular gyrus. Third, the TCM proposes a visual number form representation involved in recognizing (strings of) Arabic digits. This visual Arabic representation is supposed to be located in bilateral fusiform and lingual regions.

For more complex tasks such as multi-digit mental arithmetic, this task specificity of the different representational codes may no longer be valid. Rather, it is very likely that different numerical representations have to work in a more closely integrated manner, the more complex the arithmetic problem at hand. For instance, whenever direct fact retrieval in an arithmetic problem fails (e.g., 48+37 = ?), bilateral intraparietal areas may be involved in semantic re-coding of the problem, recruiting magnitude information of the numbers involved [6]. On the other hand, participants may also split such complex problems into simpler ones [22]. For instance, they may break down the complex problem 48+37 into more tractable pieces, such as 40+30 and 8+7, allowing assistance of magnitude-related processing by fact retrieval-related components. Therefore, the processing of complex tasks will require a close interplay of the different numerical representations described above. In turn, the distinction between different operations may not be an either/or question but a matter of degree and rather involve flexible transitions between representations.

Moreover, complex mental arithmetic has been shown to involve different and variable steps of cognitive processing, for which number specific representations are complemented by more general cognitive processes such as attention, working memory, and problem solving [23]–[25]. In particular, Dehaene and Cohen [6] suggested that the specific representations of the TCM may be further supplemented by (pre)frontal areas not necessarily specific for number processing (cf. [17]. (Pre)frontal activations may rather reflect more general cognitive processes involved in complex arithmetic. Within the frontal cortex, the dorsolateral prefrontal cortex (DLPFC), the inferior frontal gyrus, and the (pre)-supplementary motor area (SMA and pre-SMA) have been suggested to play a supportive role for multi-component cognitive functions including mathematical operations [9], [26]. In particular, the DLPFC has been associated with (amongst others) sequential ordering of operations [11], [27], working memory demands [16], [23], [27], strategic organization [11], the processing of additional operations in calculation (e.g., [16]), and rule updating of mathematical operations [28], whereas the SMA/PreSMA has been linked to processes corroborating arithmetical procedures [26].

### Numerical cognition as a case of multi-modular processing

This view is further corroborated by recent findings in functional neuroimaging. For instance, recent studies indicated a shift in brain activation from frontal to parietal regions with arithmetic training [21], [29]–[31] and age [32]. Moreover, first evidence for a flexible interplay of different numerical representations in one and the same task came from fMRI studies using a parametric modeling approach to a number bisection task [33] and to mental addition [34]. The method of parametric modeling seems to be particularly suited to investigate contributions of different representations, because it allows for defining parametric regressors representing several dimensions of complex stimuli. It allows for evaluating variations within stimulus conditions which are not due to some manipulated stimulus property (e.g., magnitude). Thus, it is possible to identify the influence of different stimulus properties in one and the same task. Following this approach, Wood and colleagues [33] were able to differentiate the influence of number magnitude, fact retrieval, and parity in a number bisection task. Moreover, Klein et al. [34] identified different effects of number magnitude processing and fact retrieval in mental addition.

In the latter study [34], participants had to indicate the correct result of single- and two-digit addition problems. The impact of number magnitude and arithmetic fact representations could be isolated by analyzing factors characterizing the stimulus set parametrically. Amongst others, parametric predictors included decade sum and unit sum with decade sum and unit sum reflecting the sum of the digits at the tens or units position of the two addends, respectively (i.e., for 23+68 decade sum equals 2+6 = 8 whereas unit sum is 3+8 = 11). In particular, the values for the predictor unit sum (which reflects the necessity of a carry operation when≥10) ranged from 3 to 17 (e.g., for 28+49, 8+9 = 17, whereas for 31+52, 1+2 = 3). When employed as a parametric predictor, it is possible to examine in which brain areas the fMRI signal is associated with increasing values of this factor (i.e., from 3 to 17 for unit sum) or rather in which brain areas the fMRI signal is associated with decreasing values of that factor (i.e., from 17 to 3). In the present case of addition, it is known that even for single-digit addition arithmetic fact knowledge can only be assumed for rather small problems (e.g., 2+3) but not for relatively larger ones (e.g., 8+9, see [35]). Applied to the case of decades and units in two-digit addition this indicates that with increasing unit and decade sum, respectively, addition problems get more difficult. This means that magnitude-related processing will be the most probable solution strategy the larger the unit and decade sum, respectively. Indeed, with increasing values of unit and decade sum Klein and colleagues [34] observed activation in brain areas associated with magnitude-related processing. By contrast, with decreasing unit and decade sum, addition problems get easier (e.g., 2+3) and may even be solved relying on arithmetic fact retrieval. In line with this argument, Klein and colleagues [34] observed activation in brain areas associated with arithmetic fact retrieval (such as the left angular gyrus) for decreasing values of unit and decade sum. These similar associations of fMRI signal with both unit sum and decade sum led us to the following idea: By looking at a conjunction of these predictors it should be possible to identify differential contributions of magnitude related processing or arithmetic fact retrieval not only for adding either tens or units, but also for the overall addition problem.

When considering the interplay of numerical and non-numerical representations it is obvious that mental arithmetic is a case of multi-modular and distributed processing. Accordingly, the TCM assumed that complex arithmetic requires the close interplay of parietal as well as additional (pre)frontal processes and the inclusion of cortical–subcortical loops involving the basal ganglia in multiplication fact retrieval [5], [6]. However, recent neuroimaging studies mainly focused on the localization of activated grey matter areas and so far no comprehensive attempt was made to investigate the connecting pathways underlying this multi-modular organisation of numerical cognition. Currently, there are only some assumptions on either general fronto-parietal connectivity [36], on parietal connectivity between different parts of the IPS and the angular gyrus [37] or a coarse functional, but non-anatomical suggestion how to integrate numerical processing into the framework of dorsal and ventral processing [38].

### The present study

In the current study we tried to address the issue of connectivity within the fronto-parietal network of numerical cognition more directly by probabilistic fiber tracking. Principally, the method of probabilistic fiber tracking was developed and is applicable to complement the anatomical specification of functionally defined networks. This means that the focus in probabilistic fiber tracking is on the identification of the most probable fiber tracts connecting the seed points of a (correctly identified) functionally defined network [39]. Basically, this is a qualitative (not quantitative) identification of the functional network in order to illustrate the most probable anatomical connections between the specified seed points. Against this background, the present study aims at identifying the connections within the associated activation patterns of the two networks of number magnitude processing and arithmetic fact retrieval in a multi-digit addition task on the group level and at integrating these connections into established frameworks of neural connectivity, such as the one differentiating dorsal and ventral processing streams. Thereby, we aim at integrating our findings on mental arithmetic with previous findings from other tracking studies in different functional contexts such as language processing. To pursue this goal we used seed regions motivated by both theoretical (TCM, [4]; [8]) as well as empirical considerations. While the former involved the cortex areas proposed to subserve either magnitude processing (IPS, PSPL), arithmetic facts (i.e., left perisylvian language areas, left AG), and visual number form representations (i.e., fusiform and lingual areas), the latter consisted of activation peaks from a re-analysis of the recent fMRI study on mental addition by Klein et al. [34]. Yet, it should be noted that the TCM does not specify coordinates for the activation of its proposed brain areas. Therefore, activation peaks from the reanalysis of the Klein et al. [34] data that corresponded to the predictions of the TCM [8] or its updates [4], were defined as theoretically based seed and target regions.

Particular interest was paid to distinguishing neural correlates and connectivity for relatively easy (conjunction of decreasing unit sum and decade sum) and more difficult (conjunction of increasing unit sum and decade sum) addition problems and their theoretically postulated, associated reliance on either verbally mediated fact retrieval or the manipulation of magnitude, respectively. Furthermore, we were interested in how far the identified networks for either, more simple or more complex addition problems can be integrated into the general distinction between dorsal and ventral processing streams. We hypothesize that processing easier problems by relying on verbally mediated arithmetic fact retrieval should draw on ventral pathways more strongly whereas more difficult problems should primarily involve dorsal pathways. In an analogy to the dorsal ‘‘where’’ and ventral ‘‘what’’ stream of the visual system [40]–[42], the distinction of ventral and dorsal processing streams has recently been extended to the domains of language [43]–[46], and spatial attention [47]. Thereby, it was possible to demonstrate the association of this dual loop system with specific functions for both language [48], [49] and attention [50]. New approaches combining the most advanced in vivo imaging techniques for the human brain have corroborated this dual stream model not only functionally but also anatomically in the human brain [51]–[53]. However, to the best of our knowledge there is so far no attempt to integrate the multi-modular organisation of numerical cognition into the framework of dorsal and ventral processing pathways. Therefore, we were interested whether the general principles associated with dorsal and ventral processing paths may be adapted to the case of mental arithmetic. Please note that more specific hypotheses on the tracking results cannot be put forward at this point because the exact seed points for the tracking were derived from a reanalysis of the Klein et al. [34] data.

To investigate the neuro-anatomical connections of the fronto-parietal network underlying mental arithmetic we evaluated the impact of a subset (unit sum and decade sum) of the individual parametric predictors included in the original analysis by Klein et al. [34]. Positive regression weights indicate concordance of predictor magnitude and level of activation (concordant activation, e.g., increasing unit sum associated with increasing fMRI signal). On the other hand, negative regression weights reflect an inverse relation between predictor magnitude and level of activation (discordant activation, e.g., decreasing unit sum associated with increasing fMRI signal). Because similar activation patterns were observed for the predictors decade sum and unit sum in our previous analysis [34], we investigated the joint impact of the two parametric predictors of interest in a conjunction analysis. A significant conjunction of concordant activations indicates areas subserving number magnitude processing, whereas, a significant conjunction of discordant activations is associated with activation in areas subserving verbally mediated processes of arithmetic fact retrieval. Accordingly, for the re-analysis of the fMRI data of Klein et al. [34] we hypothesized to find differential activation patterns. In particular, we assumed to obtain a fronto-parietal network of activation including bilateral IPS, PSPL / posterior IPS, and the DLPFC for the conjunction of increasing values of decade and unit sum, because this conjunction is expected to index increasing demands on magnitude-related processing. On the other hand, the conjunction of decreasing values of decade and unit sum should rather be associated with fact retrieval-related processing subserved by the left angular gyrus as well as left perisylvian language areas.

## Materials and Methods

To identify meaningful seed regions for the probabilistic fiber tracking we re-analyzed the fMRI data of Klein et al. [34] and compared this empirical evidence with theoretical considerations implied by the TCM [6], [8]. For a detailed description of the stimuli and the procedure of the fMRI experiment please refer to the methods section in Klein et al. [34]; for the list of all stimuli used including stimulus properties see Klein et al. [54]. The resulting seed regions were then applied to the DTI data acquired in the study by [48].

### Ethics statement

fMRI Participants were scanned with the approval of the local ethics committee of the Medical Faculty of the RWTH Aachen University; data of the DTI participants was collected with the approval of the local ethics committee of the University Medical Center Freiburg. All participants gave their written informed consent.

### Participants

In the fMRI data set 16 male right-handed healthy volunteers (mean age = 28 years; SD = 5 years) were included [34]. The DTI data was collected from a different sample of 33 healthy volunteers (11 females, mean age = 34 years, range 18–71 years, 18 right-handed) from the database of the Freiburg Brain Imaging Centre (cf. [48]). It is important to note that using independent samples of fMRI and DTI data is not a disadvantage. Instead, these independent data make it unlikely that any unknown anatomical peculiarity of a sample is decisive for the overall results, as could be the case for fMRI and DTI data from the same sample. Therefore, we are confident that using different samples for fMRI and DTI will strengthen the empirical relevance of our results, even though the two samples may not be fully comparable with respect to some demographic variables.

### Stimuli and design [34]

In a choice-reaction paradigm, 96 different single- and two-digit addition problems were presented centrally above a pair of solution probes in Arabic notation. Participants had to indicate the correct result as fast and as accurate as possible by pressing a corresponding button either with the left or the right hand. The experimental within-participant 2×2 design comprised the factors carry (carry vs. non-carry; e.g., 28+47 vs. 28+41), and (categorical) problem size (sum<40 vs.>60; e.g., 14+13 vs. 14+53).

### fMRI scanning procedure and data acquisition [34]

Functional MRI data was acquired with the Philips 1.5T Gyroscan MRI system using a standard head coil. In a rapid event-related design, 128 trials (96 experimental trials+32 null events) were presented at a rate of 4.5 s in one run, lasting about 10 minutes. Stimuli were presented using video goggles designed to meet MR requirements (http://www.mrivideo.com]). First, one functional imaging run sensitive to blood oxygenation level-dependent (BOLD) contrast was recorded for each participant (T2*-weighted echo-planar sequence, TR = 2800 ms; TE = 50 ms; flip angle = 90°; FOV = 240 mm, 64×64 matrix; 30 slices, voxel size = 3.75×3.75×4 mm^3^). Additionally, 4 dummy scans were acquired to allow for steady magnetization. Second, for each participant, a high-resolution T1-weighted anatomical scan was acquired (TR = 30 ms; TE = 4.6 ms; flip angle = 30°; FOV = 256 mm; 256×256 matrix; 160 slices; voxel size = 1×1×1 mm^3^).

### DTI scanning procedure and data acquisition [48]

In vivo DTI-measurements were performed on a 3T whole body MR system (TIM Trio, Siemens, Erlangen, Germany) using a standard circularly polarised radio frequency 12-channel head coil with a diffusion-weighted Spin-Echo EPI sequence. The whole brain was covered with contiguous 2 mm slices with an in-plane resolution of 2×2 mm^2^ Diffusion encoding was performed in 61 different directions with an effective b-value of 1000 s/mm^2^. Distortion correction was applied according to Zaitsev et al. [55]. For each participant of the DTI sample, a high-resolution T1-weighted anatomical scan was acquired (TR = 2200 ms; TE = 2.15 ms; flip angle = 12°; FOV = 256 mm; 256×256 matrix; 160 slices; voxel size = 1×1×1 mm^3^).

**Analysis.** Behavioural and imaging analyses were based on correct trials only, resulting in a loss of 8.4% of the data. Furthermore, items with response latencies falling outside the interval between 200 ms and 3500 ms were not considered. In a second step, responses outside the interval of +/-3 standard deviations around the individual mean were excluded. An additional 0.26% of the data was excluded due to this trimming procedure.

Behavioural data of the study by Klein et al. [34] were reanalysed, conducting a multiple regression analysis on mean item RT and comprising the predictors decade sum and unit sum. We chose these two predictors because the fMRI analysis is based on a conjunction of the parametric analysis of these two predictors. It should be noted, however, that, in contrast to the parametric analysis of imaging data, it is not possible to distinguish between effects for increasing or decreasing values of the predictors in the behavioral data. As there were too many perfect scores, no regression on error rates was conducted.

Anatomical scans for both fMRI and DTI samples were segmentation-based normalized and averaged in SPM8 (http://www.fil.ion.ucl.ac.uk/spm). The fMRI time series were corrected for movement artifacts and unwarped in SPM8. Images were motion corrected and realigned to each participant’s first image. Data were normalized into standard stereotaxic MNI (Montreal Neurological Institute) coordinates space. Images were resampled every 3.75 mm using trilinear interpolation and smoothed with a 7.5 mm FWHM Gaussian kernel to accommodate inter-subject variation in brain anatomy and to increase signal-to-noise ratio in the images. The data were high-pass filtered (128 s) to remove low-frequency signal drifts and corrected for autocorrelation assuming an AR(1) process. Brain activity was convolved over all experimental trials with the canonical hemodynamic response function (HRF). Onsets of incorrectly answered trials were entered separately as a condition of no interest into the model.

In the study of Klein et al. [34], amongst others the impact of parametric predictors representing decade sum and unit sum were estimated in a parametric analysis. For the re-analysis of this data, we focused on a conjunction of the two predictors decade sum and unit sum. For the conjunction we first calculated the t-maps within SPM 8 separately for both the influence of decade sum and unit sum. Then the conjunction of these two contrasts was calculated. Decade sum and unit sum reflect the sum of the digits at the decade or unit position of the two addends, respectively (i.e., for 23+68 decade sum is 2+6 = 8 while unit sum is 3+8 = 11). For both predictors increasing values were associated with an increase of activation in areas subserving magnitude-related processing (e.g., IPS, PSPL), whereas decreasing values were associated with increasing activation in areas assumed to be recruited in arithmetic fact retrieval (e.g., left AG). Therefore, we conducted the conjunction of the impact of predictors decade sum and unit sum. A significant conjunction of concordant activations of these predictors should indicate areas subserving number magnitude processing, whereas a significant conjunction of discordant activations should be associated with activation in areas subserving verbally mediated processes of arithmetic fact retrieval. The peak voxels of each contrast were identified separately at a statistical threshold of p<.005 per voxel with a cluster size of k = 10 voxels. However, it should be noted that our results exclusively stem from conjunctions of such contrasts, which tend to retain only stable aspects of the activation patterns.

For the anatomical localisation of effects, we used the SPM Anatomy Toolbox [56], available for all published cytoarchitectonic maps from www.fz-juelich.de/ime/spm_anatomy_toolbox as well as the anatomical automatic labeling tool (AAL) in SPM8 (http://www.cyceron.fr/web/aal_anatomical_ automatic_labeling.html).

### Definition of seed regions

Seed regions for the probabilistic fiber tracking were extracted in MNI space from the second-level t-maps of the parametric reanalyses of the fMRI data by Klein et al. [34]. Primary and secondary peak voxels were identified within the activation clusters. In a next step, the DTI data from the database of the Freiburg Brain Imaging Centre were used (cf. [48]). In particular, all of the coordinates identified in the re-analysis of Klein et al. [34] were transferred to the native space of each participants DTI data from Saur et al. [48] using the inverse normalization parameters obtained from the segmentation procedure of the T1 anatomical scan and enlarged to a sphere with a radius of 7 mm, each containing 33 seed voxels. These spheres defined the seed regions for the probabilistic fibre tracking procedure.

### Probabilistic DTI-based fiber tracking

DTI data were analyzed using the method of pathway extraction developed by Kreher et al. [39, see also 48 for a similar procedure] of pathway extraction, which is implemented in the Matlab-based DTI and Fiber Toolbox (www.uniklinik-freiburg.de/mr/live/arbeitsgruppen/diffusion/fibertools_en.html).

The diffusion tensor (DT) was calculated in a first step from the movement- and distortion-corrected diffusion-weighted imaging dataset [57]. Subsequently, probabilistic maps were calculated for each seed region by means of a Monte Carlo simulation of Random Walks similar to the Probabilistic Index of Connectivity (PICo) method [58]. The tracking procedure differed from the classical PICo method by empirically extracting the orientation density function from the diffusion tensor and by preserving the main traversing direction (in relation to the first Eigenvector) of each propagated trajectory during the random walk ( =  extended probabilistic tracking, see [39] for details). This directional information is important for the subsequent multiplication procedure (see below). In these maps, the visiting frequency of a voxel reflects the degree of connectivity to the seed region. Following the procedure of Kreher et al. [39], the number of random walks was set to 10^5^ and maximum fiber length to 150 voxels. Tracking area was restricted to a white-matter mask to avoid tracking across anatomical borders. To ensure contact of the cortical seed regions with white matter, a rim of gray matter was included in the mask. Third, region-to-region anatomical connectivity between two seed regions (A and B) was computed using a newly developed combination of probability maps [39]. Computationally, this combination implies two voxel-wise vector multiplications of the visiting maps, which take into account both the frequency of visits from both seeds in a given voxel and the direction of the random walks. First the product of the two visiting maps from the seed region and from the target region is calculated. Second, this intersection is again multiplied by a map coding the relative travel direction between the two visiting maps. This second step eliminates all regions where the relative travel direction is parallel, meaning that both fibers run to a third region, and keeps all regions where the relative direction is opposite. In particular, *w*alks starting from two seed regions A and B may either face in opposing directions (connecting fibers) or they merge and point in the same direction (merging fibers). Within the pathway connecting both seeds, the proportion of connecting fibers should exceed the proportion of merging fibers. Using the directional information (obtained by extended PICo) during the multiplication procedure, merging fibers are suppressed, while connecting fibers are preserved [39]. Thereby, this measure is based on two constraints: (i) Each voxel has to be connected to both seed regions A and B, thus only voxels which are effectively reached from both seeds constitute the connection of interest. (ii) Trajectories started in seed region A have to run in opposite direction to those started in B. In contrast, connections where trajectories have the same directionality (e.g., within a diverging connection to a third region C are suppressed (see [39] for details). This method enables the extraction of the most probable direct pathway between two seed regions without using a priori knowledge about the presumed course. In the resulting combined maps, values represent a voxelwise estimate of the probability index that a voxel is part of the connecting fiber bundle of interest (probability index on forming part of the bundle of interest, PIBI). Taken together, this method (i) enables a sensitive identification of region-to-region connections without a priori knowledge about its course and (ii) ensures that only direct anatomical connections between two seed regions are considered.

### Postprocessing of probability maps

Using SPM8 (www.fil.ion.ucl.ac.uk/spm) the combined maps of the previously 10^5^ send out individual samples were scaled to the range 0–1 and spatially normalized into standard MNI space.

Group maps for each region-to-region connection were computed by averaging the combined maps from all participants. Consequently, voxels represent the arithmetic mean of the PIBI from all contributing probability maps. To remove random artefacts, only voxels with PIBI values>0.0148 were displayed, which excludes 95% of the voxels with PIBI >10^-6^, guaranteeing that only the highest 5% of PIBI were considered. This value was generated empirically from the distribution observed in a large collection of pre-processed combined probability maps [39]. Please note that in this way for all individual PIBIs the same threshold of>0148 was applied, ensuring all fibres displayed are thresholded at the same level. 3D volume renderings of pathways were visualized with an in-house application from the Freiburg Brain Imaging Center based on the free software OpenDX (http://www.opendx.org). Tracking results were derived from publications on connectivity of macaque cortex [59] as well as the human cortex [60].

## Results

### Behavioural data

Overall mean RT was 1972 ms with a standard deviation of 497 ms. We conducted a multiple regression analysis incorporating the predictors decade sum and unit sum. In this model (R = .80, R^2^ = .65, adjusted R^2^ = .64, *F*(2, 93) = 84.5, *p*<001) both predictors were significant (both *p*<001). Closer inspection of the beta values indicated that RT increased with increasing decade sum (b = .79) as well as increasing unit sum (b = .33). The respective regression equation was as follows: RT = 985 ms+121 ms * decade sum+48 ms * unit sum. In absolute this means that an increase of one in decade sum increased RT by 121 ms, whereas a comparable increase in unit sum increased RT by 48 ms only.

### fMRI data

#### Conjunction of increasing decade sum and increasing unit sum

**Figure 1:**
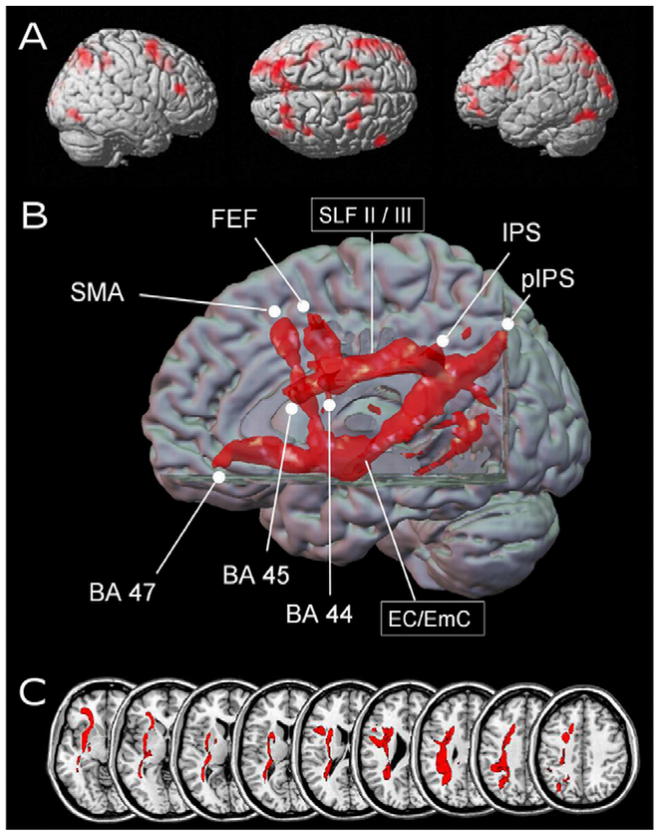
Results for more difficult mental arithmetic (e.g., 54+38 = ?). Panel A reflects cortical regions showing increase of fMRI signal due to *increasing* values of the conjunction of unit sum as well as decade sum (uncorrected p-value of<.005, cluster size k = 10 voxels). Activated areas for magnitude-related processing include IPS, pIPS, BA 44, BA 45, BA 47, SMA, FEF, and the visual number form (VNF), most of them bilaterally. Panel B depicts identified pathways in a 3D volume rendering with PIBI values>0.0148. It can be observed that for more difficult mental arithmetic a system of both dorsal (SLF) and ventral (EC/emC) connections is recruited (depicted in red). Panel C gives a detailed view on the course of the fiber tracts in axial orientation (depicted in red), demonstrating a dorsal vs. ventral fiber pathway profile, encompassing the SLF system and the EC/EmC system. Abbreviations: BA = Brodmann area; EC = external capsule; EmC = extreme capsule; FEF = frontal eye fields; IPS = intraparietal sulcus; pIPS = posterior intraparietal sulcus; SLF = superior longitudinal fascicle; SMA = supplementary motor area.

 The fMRI signal was modulated significantly by the joint prediction of *increasing* decade and unit sum in the bilateral intraparietal sulci (BA 7) and the bilateral posterior intraparietal sulci (BA 7) at an uncorrected p-value<.005 and for a cluster size of k = 10 (see Panel A of Figure 1 and Table 1). Further clusters of activated voxels were observed in the left inferior frontal gyrus in BA 45, BA 44, and BA 47 as well as in the right inferior frontal gyrus (BA 45). Moreover, activation was found in the bilateral supplementary motor areas (SMA, BA 6) and the bilateral middle frontal gyri (BA 6), possibly situating the bilateral frontal eye fields (FEF). Additionally, activation was observed in the left fusiform gyrus (BA 19), possibly indicating activation of the visual number form (VNF) area and visual cortices, bilaterally (BA 18).

**Table 1:** Cortical regions activated significantly more due to either the conjunction of increasing or decreasing unit sum and decade sum.

	Brain region (BA)	MNI (x, y, z)			Cluster size	Z score
**Conjunction Increasing decade sum, unit sum**	LH intraparietal sulcus (BA 7)	−45	−38	45	35	3.43
	RH intraparietal sulcus (BA 7)	41	−49	45	14	3.10
	LH posterior intraparietal sulcus (BA 7)	−30	−71	49	62	3.46
	RH superior parietale lobule (BA 7)	19	−68	56	57	3.93
	LH inferior frontal gyrus (BA 45)	−53	19	26	95	3.75
	RH inferior frontal gyrus (BA 45)	53	34	19	15	3.66
	LH inferior frontal gyrus (BA 44)	−49	11	26	95	3.75
	LH inferior frontal gyrus (BA 47)	−36	45	−15	23	3.10
	LH supplementary motor area (BA 6)	−9	19	45	52	4.07
	RH supplementary motor area (BA 6)	9	4	68	12	3.06
	LH middle frontal gyrus (BA 6)	−26	7	55	34	3.22
	RH middle frontal gyrus (BA 6)	30	7	55	32	3.02
	LH fusiform gyrus (BA 19)	−38	−71	−15	44	3.62
	LH middle occipital gyrus (BA 18)	−26	−98	15	52	3.66
	RH inferior occipital gyrus (BA 18)	45	−79	−4	12	3.33
**Decreasing decade sum, unit sum**	LH angular gyrus (BA 39)	−53	−64	23	104	3.20
	RH angular gyrus (BA 39)	60	−53	34	121	3.19
	LH supramarginal gyrus (BA 40)	−64	−24	23	50	2.91
	RH supramarginal gyrus (BA 40)	64	−45	38	34	2.81
	LH middle temporal gyrus (BA 39)	−60	−60	8	104	3.20
	RH middle temporal gyrus (BA 39)	53	−16	19	23	3.38
	LH superior temporal gyurs (BA 41)	−56	−23	11	20	2.35
	LH insula	−38	4	−4	57	2.78
	LH retrosplenial cortex (BA 31)	−9	−53	30	94	2.64
	RH retrosplenial cortex (BA 31)	9	−49	30	94	3.13
	LH medial frontal gyrus (BA 32)	−9	45	11	184	3.70
	RH medial frontal gyrus (BA 32)	9	49	−15	72	3.21
	LH hippocampus	−23	−15	−26	11	2.47

p<.005, uncorrected; cluster size = 10 voxels; MNI: Montreal Neurological Institute coordinates.

#### Conjunction of decreasing decade sum and decreasing unit sum

**Figure 2:**
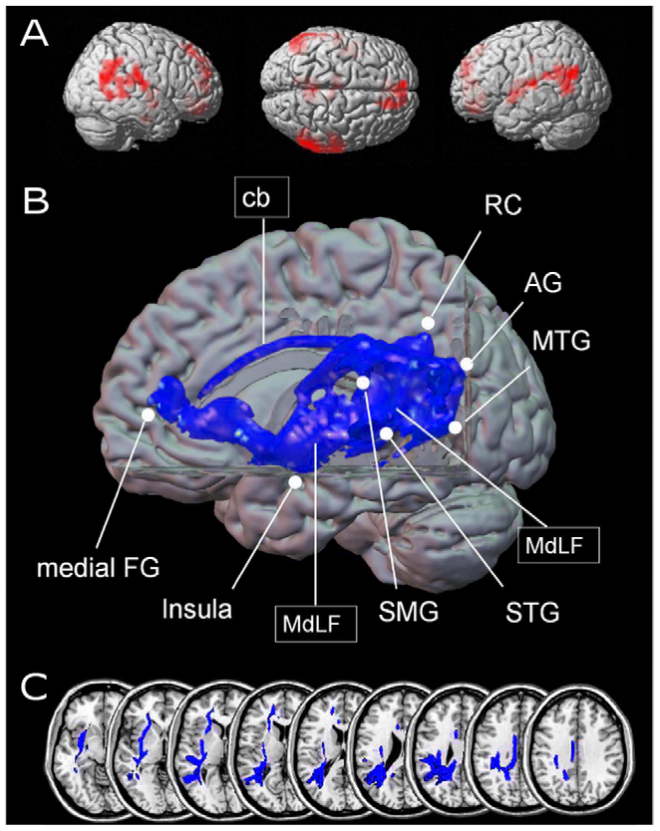
Results for relatively easier mental arithmetic (e.g., 14+3 = ?). Panel A reflects cortical regions showing increase of fMRI signal due to *decreasing* values of the conjunction of unit sum as well as decade sum (uncorrected p-value of<.005, cluster size k = 10 voxels). Activated areas for fact-retrieval related processing include left AG, STG, MTG, insula, retro-splenial cortex (RC), and medial FG. Panel B shows identified pathways for easier mental arithmetic in a 3D volume rendering (PIBI values>0.0148) indicating prominently ventral (MdLF) connections (depicted in green). Furthermore, a dorsal connection via the cingulate bundle was tracked linking retro-splenial cortex with the medial frontal gyrus. Panel C again gives a detailed view on the course of the fiber tracts in axial orientation illustrating the prominently ventral fiber pathway profile. The ventral connection encompassed the MdLF and the more superior and medial part of the EC/EmC system (depicted in green). Abbreviations: AG = angular gyrus; cb = callosal bundle; medial FG = medial frontal gyrus; MdLF = middle longitudinal fascicle; MTG = middle temporal gyrus; RC = retrosplenial cortex; SMG = supramarginal gyrus; STG = superior temporal gyrus.

 The joint prediction of *decreasing* decade sum and unit sum was associated with fMRI signal change (uncorrected p-value<.005, cluster size k = 10) in the bilateral angular gyri (BA 39), the bilateral supramarginal gyri (BA 40), and the bilateral middle temporal gyrus (BA 39). Further clusters of activated voxels were observed in left perisylvian language areas such as the left superior temporal gyrus (BA 41) and the left insula (see Panel A of Figure 2 and Table 1). Additionally, activation was observed in the bilateral retrosplenial cortices (BA 31), the bilateral medial frontal gyri (BA 32), and, importantly, the left hippocampus possibly indicating activation related to the processing of familiar objects and procedures and to retrieval from long-term memory.

Seed regions. To define meaningful seed regions we compared the empirical evidence from the reanalyzed fMRI data with theoretical considerations implied by the TCM and its latest amendments with respect to mental arithmetic [4], [6], [8]. In particular, for difficult mental arithmetic the TCM proposes bilateral activation of the IPS, the PSPL/pIPS, and the fusiform gyrus, which were all found in the re-analysis of Klein et al. [34] and, therefore, included as seed regions for fiber tracking (Table 2). Furthermore, the TCM assumes that frontal areas, which are not specified in more detail, are recruited in difficult mental arithmetic. In the re-analysis of the Klein et al [34] data we were able to identify the following frontal regions, which were incorporated as seed regions: BA 44, BA 45, BA 47, and SMA (the latter was already recommended by Arsalidou et al. [4]). For easier mental arithmetic the TCM proposes activation of the left AG and of left-hemispheric perisylvian language areas, which we also found in the re-analysis of the Klein et al. [34] data and which we incorporated as seed regions (left AG, SMG, STG, MTG, and the insula).

**Table 2:** Seed regions and connections included. Seed regions were centered at the MNI coordinates given in parentheses.

	Seed		Target	Relative frequency of individual tracts accounting for mean tract
**More Difficult (magnitude-related) Mental Arithmetic**	LH IPS [−45 −38 45]	↔	LH pIPS [−30 −71 49]	33/33
	LH IPS [−45 −38 45]	↔	LH IFG (BA 45) [−53 19 26]	33/33
	LH IPS [−45 −38 45]	↔	LH IFG (BA 44) [−49 11 26]	33/33
	LH IPS [−45 −38 45]	↔	LH IFG (BA 47) [−36 45 −15]	31/33
	LH IPS [−45 −38 45]	↔	LH SMA [ −9 19 45]	32/33
	LH pIPS [−30 −71 49]**	↔	LH FEF [−26 7 55]**	31/33
	LH VNF [−38 −71 −15]	↔	LH IPS [−45 −38 45]	29/33
**Easier (verbally mediated) Mental Arithmetic**	LH AG [−53 −64 23]	↔	LH SMG [−64 −24 23]	28/33
	LH AG [−53 −64 23]	↔	LH MTG [−60 −60 8]	33/33
	LH AG [−53 −64 23]	↔	LH STG [−56 −23 11]	31/33
	LH AG [−53 −64 23]	↔	LH insula [−38 4 −4]	33/33
	LH AG [−53 −64 23]	↔	LH RC [ −9 −53 30]*	29/33
	LH AG [−53 −64 23]	↔	LH medial FG [ −9 45 11]*	31/33
**Interhemispheric tracking**	LH IPS [−45 −38 45]	↔	LH pIPS [−30 −71 49]	33/33
	LH IPS [−45 −38 45]	↔	RH IPS [ 41 −49 45]	30/33
	RH IPS [ 41 −49 45]	↔	RH PSPL [19 −68 56]	33/33

AG: angular gyrus; BA: Brodmann Area; FEF: frontal eye fields; IFG: inferior frontal gyrus; IPS: intraparietal sulcus; LH: left hemisphere; medial FG: medial frontal gyrus; MTG: middle temporal gyrus; pIPS: posterior intraparietal sulcus; PSPL: posterior superior parietal lobule; SMA: supplementary motor area; SMG: supramarginal gyrus; STG; superior temporal gyrus; RC: retrosplenial cortex; RH: right hemisphere; VNF: visual number form area.

* Seed points proposed but not exactly specified by the TCM are given in italics. Second, the seed points not proposed by the TCM but nevertheless theoretically motivated are given in italics and marked by an asterisk.

** Please note that the fiber pathways involved travel from the FEF first via the LH IPS before arching in association fibres to the pIPS (thereby making the connection between FEF and pIPS almost identical to the connection between FEF and IPS).

Number of individual tracts accounting for mean tract:

Evaluation of how consistently the mean-courses of the tracts are reflected by the individual tracts of the participants. This way, it is possible to indicate, in how many participants their individual tracts are more or less identical to the mean tracts. However, for those participants whose individual tracts does not correspond to the mean course of the respective tract, it is important to note that the respective PIBI value can nevertheless be *large* (simply reflecting that the algorithm can tell for sure that the respective tract passes via a different pathway in these participants).

Nevertheless, this means that the mean tracts identified by our probabilistic fibertracking accounted for at least 28 out of 33 individual tracts.

Furthermore, we had specific hypotheses which have not yet been specified anatomically for the TCM. Therefore, we added further seed regions indicated by our re-analysis of the Klein et al. [34] data. For *arithmetic fact retrieval*, this approach led to the inclusion of the retrosplenial cortex as well as the medial frontal gyrus. The reason for this was that the retrosplenial cortex in connection with the medial frontal gyrus has been observed repeatedly to be involved in the recognition of familiar objects and procedures (e.g., [61]), which corresponds well functionally l to the retrieval of familiar arithmetic facts from memory on a theoretical level. On an empirical level, this area has also been found repeatedly to contribute to mental arithmetic and to connect the angular gyrus with the hippocampus [61] and to the processing of overlearned arithmetic facts (e.g., [62]). Moreover, for *number magnitude related processing*, the frontal eye fields (FEF) were added (as previously recommended by [4]). In 2009, Knops et al. were able to show that the PSPL (associated with orientation on the mental number line) is also involved in saccadic eye-movements [63]. More importantly, the results suggested that the pIPS/PSPL is associated with the parietal eye fields (PEF). Since the frontal eye fields are directly and semantically connected to the parietal eye fields and were found to be activated in the reanalysis of the Klein et al. [34] data, the FEF were included as seedpoints as well (Table 2).

Since (i) the main regions supposed to be involved in the verbal system are located in the left hemisphere and (ii) the main regions assumed to contribute to magnitude processing are supposed to be situated bi-hemispherically (which is also true for the visual number form area when Arabic digits are involved), we focus on our trackings for the left hemisphere. Please note that two different functional networks, which do not share a seed point, are actually the *necessary* prerequisite for probabilistic fiber tracking. Because both the IPS as well as the AG are connected to nearly all brain regions [64], the fact that the two networks consist of different seed points does not preclude that the networks share fiber tracts.

### DTI data

Fiber tracts connecting frontal and parietal regions in difficult mental arithmetic. Seed regions derived from the conjunction analysis indicating magnitude-related processing (see Figure 1A, Panel A) were connected by both dorsal streams, corresponding to the superior longitudinal fascicle (SLF), as well as ventral streams, corresponding to the more inferior and lateral part of the extreme and/or external capsule system (EC/EmC). In particular, the majority of fiber tracts from the supplementary motor area, the frontal eye fields (middle frontal gyrus), and the IFG (BA 44 and BA 45) run in lateral and medio-dorsal (SLF III and SLFII) parts of the SLF towards the parietal seedpoints IPS and pIPS. However, obviously also ventral connections were observed. Rostrally, the ventral pathways ran laterally and inferior to the external/extreme capsule and continued towards BA 47 of the IFG. Moreover, some connections emerging from the rostral seeds of the IFG (BA 45) additionally showed sparse fibers running through the extreme capsule at a different ventral position (Figure 1, Panels B and C).

Fiber tracts connecting frontal and parietal regions in easy mental arithmetic. We found a predominance of ventral fibers (Figure 2B) between fronto-parietal areas involved in the retrieval of arithmetic facts. The STG, insula, MTG, and SMG appeared to be connected entirely via the ventral route. The fiber course shows considerable anatomical consistency across the middle longitudinal fascicle (MdLF) and converges into the subinsular white matter near the claustrum; it is assigned to the more superior and medial part of the extreme and/or external capsule (EC/EmC) as compared to the fibers involved in magnitude-related processing. Furthermore, both, a dorsal connection via the cingulate bundle but also a ventral pathway was observed, linking the retrosplenial cortex with the medial frontal gyrus, which has repeatedly been associated with the recognition of familiar objects or procedures.

Nevertheless, the ventrally oriented MdLF fibers system clearly is the main fiber pathway connecting frontal and parietal areas of the network in fact retrieval.

**Figure 3:**
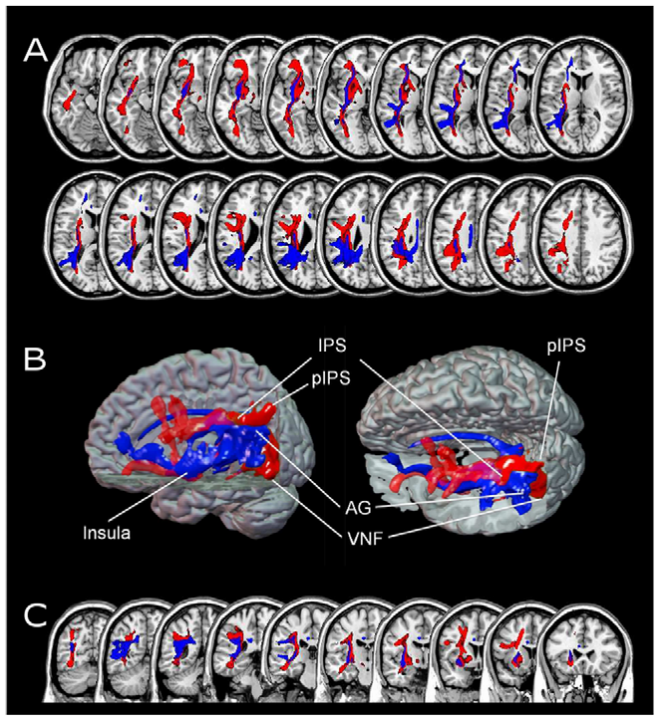
Overlay of fiber tracts identified for more difficult (red) and easier (blue) mental arithmetic. Panel A gives a detailed view on the course of the fiber tracts in axial orientation. Two largely anatomically distinct dorsal vs. ventral fiber pathway profiles for the processing of more difficult (magnitude-related) and the easier (fact retrieval related) problems can be observed. Importantly, difficult mental arithmetic (depicted in red) and easier mental arithmetic (depicted in blue) seems to involve two largely separate networks differing not only in localization of activation but also in the connections between associated cortex areas. Additionally, the connection between the visual number form area (VNF) and the number magnitude representation (IPS/pIPS) is displayed in red. Panel B again reflects the identified pathways in a 3D volume rendering with PIBI values>0.0148. Finally, Panel C depicts a detailed view on the course of the fiber tracts in coronal orientation, illustrating that easier mental arithmetic encompasses the EC/EmC system more superiorly and medially (depicted in blue), whereas more difficult mental arithmetic recruits more inferior and lateral components of the EC/EmC system (depicted in red).Abbreviations: AG = angular gyrus; IPS = intraparietal sulcus; pIPS = posterior intraparietal sulcus; VNF = visual number form.

Overlay of magnitude- and fact retrieval-related processing. Evaluation of the overlay of rather difficult and rather easy mental arithmetic suggests that magnitude- and fact retrieval-related processing seem to be subserved by two largely separate networks; these networks not only differ in localization of activation but also in the connections between cortex areas involved. Figure 3 illustrates from three different views (A: axial slices, B: 3D presentation, C: coronar slices) that both networks involve dorsal and ventral pathways but only visually overlap to a very limited degree in Figure 3 Panel B. However, because white matter tracts are usually covered with myelin, this means that (visually) overlapping tracts as revealed by pink colour do not necessarily indicate contact between the two networks Nevertheless, it is important to note that both networks, even though seemingly distinct anatomically, work as a functionally integrated circuitry for mental calculation, because they were identified applying parametric analyses for the same task. Please note that Figure 3 also shows the connection from the visual number form area in the fusiform gyrus to the left IPS, representing the connection from the visual code to the number magnitude code. This connection is arching around the occipital horn of the lateral ventricle and probably sharing fibers with the inferior longitudinal fascicule (ILF), the inferior fronto-occipital fascicle (IOF) and/or the posterior parts of the posterior thalamic radiation.

In summary, for difficult (i.e., primarily magnitude-related) calculation, areas in intraparietal cortices (IPS, PSPL) assumed to subserve magnitude manipulations are connected to premotor and frontal areas via both dorsal and ventral pathways. In particular, the dorsal connection (via the superior longitudinal fascicule system) to BA 44 may reflect processes of verbal working memory, while the ventral pathway (via the extreme capsule and/or the claustrum) to BA 45 and BA 47 may be associated with the application of rules.

For relatively easier (i.e., more fact retrieval-related) calculation, areas associated with arithmetic fact retrieval (e.g., the AG) were more prominently connected to temporal and frontal areas via the ventral stream (e.g., via the MdLF), possibly indicating phonological/semantic access. Nevertheless, a dorsal connection (via the callosal bundle) was observed as well between areas associated e. g. with the recognition of familiar objects (for a review see [61]).

#### Bi-hemispheric representation of number magnitude

**Figure 4:**
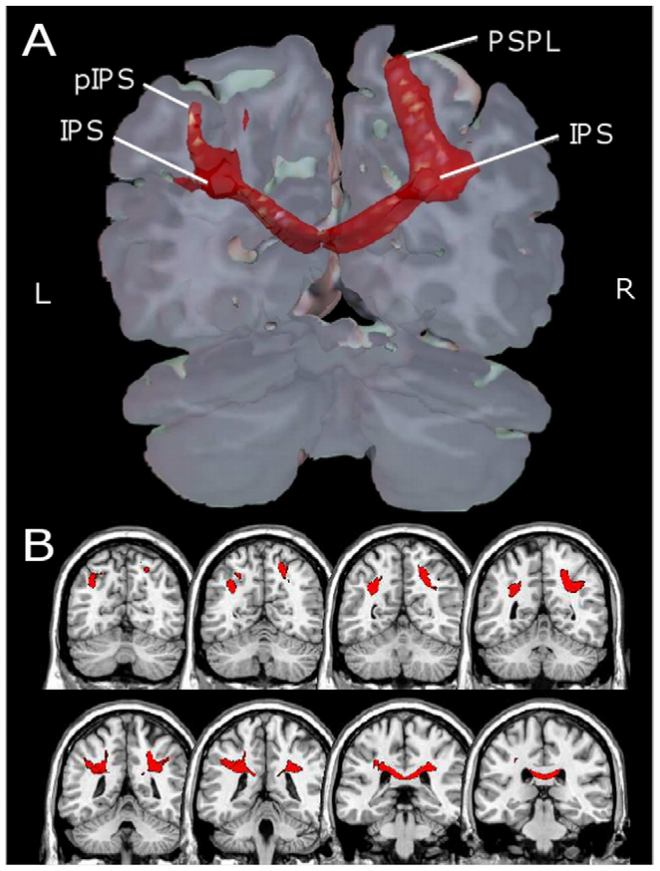
Inter-hemispheric tracking of the connection of the bi-hemispheric representation of number magnitude in intraparietal cortices. Panel A depicts the transcallosal bundle of fibers directly connecting left and right intraparietal cortices in a 3D volume rendering with PIBI values>0.0148. Panel B gives a detailed view on the course of the fiber tracts in coronal orientation.Abbreviations: IPS = intraparietal sulcus; pIPS = posterior intraparietal sulcus; PSPL = posterior superior parietale lobule.

 Central to the number magnitude representation assumed by the TCM is the notion that quantity information is represented redundantly in both hemispheres of the human brain within the intraparietal cortices. These bilateral intraparietal cortices are suggested to be connected through trans-callosal fibers, which enable the interplay between the left and the right hemisphere [13], [14]. Nevertheless, due to practical reasons in probabilistic DTI-based fiber tracking, we focused our trackings on only one hemisphere. As the TCM suggests the verbally mediated processing of arithmetic facts to be left-lateralized, we chose the left hemisphere for this purpose. However, because of the theory-based assumption of a bi-hemispheric number magnitude representation, we were also interested in demonstrating inter-hemispheric tracking of the bilateral IPS and posterior intraparietal structures. And indeed, as depicted in Figure 4, the trans-callosal bundle of fibers directly connected left and right intraparietal cortices. This finding is important as it corroborates one central notion of the TCM [8] that number magnitude information is represented bilaterally. Additionally, validating this TCM prediction further corroborates the general validity of the current probabilistic fiber tracking approach.

## Discussion

In the current study, we were interested in whether there exist different processing pathways for easier (verbally mediated) and more difficult (magnitude-related) mental arithmetic within the proposed fronto-parietal network for numerical cognition. With respect to the fiber connections between the cortex areas involved, we hypothesized that general principles associated with dorsal and ventral processing paths, as identified in studies on visual [40], [42] or attentional processing [50], may be adapted to the case of mental arithmetic. In particular, we hypothesized that for relatively easier (i.e., fact retrieval related) mental arithmetic the distribution of activated cortex areas should be prominently connected by ventral pathways, while for more difficult (i.e., magnitude-related) mental arithmetic connections within the fronto-parietal network should prominently follow dorsal pathways. To evaluate these hypotheses, we jointly considered empirical evidence from a re-analysis of fMRI data of the study by Klein et al. [34] and theoretical considerations implied by the TCM and its latest amendments for the definition of seed points required for an analysis of fiber tracts [4], [6], [8].

Indeed, we were able to identify two networks for the processing of difficult mental arithmetic on the one hand and easier mental arithmetic on the other hand, which were largely distinct from an anatomical point of view. Importantly, this becomes evident when evaluating both (i) the fMRI activation peaks as well as (ii) the fiber tracking results.

First, in a parametric analysis we were interested in what way smaller values of tens and unit digits, associated with more retrieval related verbally mediated solutions, and relatively larger values of tens and units, associated with magnitude related solutions, predicted fMRI signal change. As regards the activation peaks revealed by this re-analysis of the Klein et al. [34] fMRI data, we observed a clear-cut difference for magnitude-related and verbally mediated processing. On the one hand, for difficult (i.e., primarily magnitude-related) mental arithmetic we observed a fronto-parietal network comprising magnitude-related activation of the bilateral intraparietal cortices (IPS, PSPL) as well as frontal activation related to working memory (e.g., BA 44), the application of rules (BA 47) and processes supporting operation procedures (SMA). On the other hand, for relatively easier (i.e., more fact retrieval-related) mental arithmetic we observed areas associated with arithmetic fact retrieval (e.g., the left AG and left-hemispheric language areas) and the recognition of familiarity (e.g., retro-splenial cortex). Importantly, it should be noted that we observed significant concordant and discordant fMRI changes. This means that we did not only find generally more activation in magnitude-related mental arithmetic. Instead, we found different localizations of the activation peaks for verbally mediated and magnitude related mental arithmetic. This means that the possible difference between the underlying networks may reflect qualitative processing differences rather than a merely quantitative effect of task difficulty.

Second, this interpretation of qualitative processing differences was further substantiated by the fiber tracking results. Again, qualitative differences between the two networks became evident since the connections within each network run in largely distinct anatomical pathways. Although both magnitude-related processing as well as verbally mediated processing recruited both dorsal and ventral fiber tracts, the dorsal and ventral pathways were not identical but largely anatomically distinct. In particular, for magnitude-related processing, the dorsal pathways involved the SLF-system (SLF II and III) for the connection of the intraparietal cortex with frontal areas, whereas for verbally mediated processing a dorsal connection via the cingulate bundle was observed connecting the retro-splenial cortex with the medial frontal cortex [65]. As regards the ventral pathways, in difficult mental arithmetic the EC/EmC system was involved (connecting the intraparietal cortex and the inferior frontal cortex (BA 47)); however, the connections observed encompassed the EC/EmC system more inferiorly and laterally than in easier mental arithmetic. Additionally, in easier mental arithmetic the parietal angular gyrus was connected via the MdLF with perisylvian language areas (SMG, STG, MTG, insula), and the medial frontal gyrus.

Taken together, this means that mental arithmetic is not carried out along either dorsal or ventral pathways, depending on whether it is performed prominently by magnitude manipulations of arithmetic fact retrieval, respectively. Instead, in both cases both dorsal and ventral pathways were involved. These findings suggest that the distinction between rather magnitude-related processing and rather verbally mediated processing may not be a question of either/or but rather indicate that representations of number magnitude information and arithmetic facts are recruited concomitantly. Moreover, involvement of and connections to frontal cortex areas further suggest that these number-specific representations are complemented by more general cognitive processes such as working memory [22]–[24], cognitive control (e.g., [66]), and rule updating of mathematical operations [27] not specific to the number domain, as identified by the current fiber tracking results. Thereby, the current study is the first to specify the anatomo-functional pathways connecting the fronto-parietal cortex areas assumed to underlie numerical cognition according to the TCM.

In the remainder of the discussion we would like to comment on four additional aspects, which may not be related to our research question directly but, nevertheless, have important implications for the anatomo-functional specification of the TCM. This is (i) representational characteristics of arithmetic fact knowledge, (ii) the retro-splenial cortex in arithmetic fact retrieval, (iii) the interplay between the magnitude- and fact retrieval related network, and (iv) the fact that the number magnitude representation is assumed to be bi-hemispheric [8]. Finally, we will offer an interpretation of the current data in terms of the dual loop model [49].

### Interplay between magnitude- and fact retrieval related network

It is important to note that both networks are seemingly distinct anatomically. Fiber systems connecting number magnitude processing (associated with IPS and PSPL activation) with additional frontal (non-numerical) areas subserving more general cognitive processes such as working memory and cognitive control were found to be different from fiber systems connecting verbally mediated retrieval of arithmetic facts (left AG) with perisylvian language areas. This finding delineates significant evidence for a clear-cut distinction between the processes involved in mental arithmetic, such as arithmetic fact retrieval on the one hand and arithmetical procedures and strategies involving magnitude manipulations on the other. However, as both of these networks were identified applying the same parametric predictors in an analysis of the same task also suggests that they work as a functionally integrated circuit for mental arithmetic. Thus, the two networks seem to operate in a closely integrated fashion but the exact way in which the two networks work together and the question *through which anatomical structures* this cooperation is characterized awaits further clarification.

**Figure 5:**
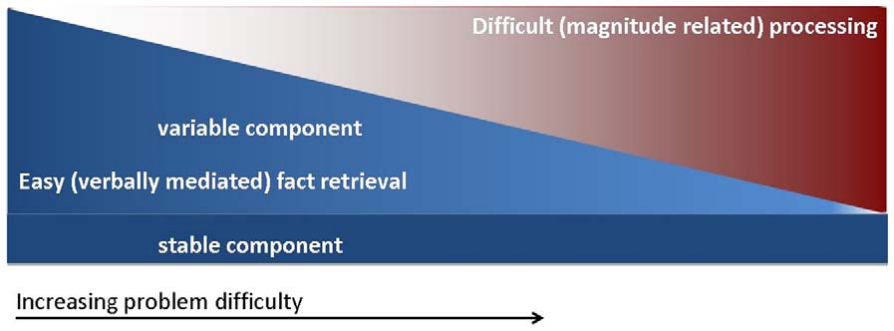
Hypothetical model capturing the interplay between magnitude-related processing and arithmetic fact retrieval. The figure depicts an interaction model: Basic fact retrieval related processing is assumed to be involved even in difficult arithmetic, for instance to retrieve partial results from long term memory. However, in easier tasks a larger variable component of fact retrieval may be recruited, whereas in more difficult tasks more and more magnitude-related processing is assumed.

In the following, we suggest such a hypothetical model (cf. Figure 5). In this model, a stable influence of fact retrieval processes is assumed on both easier and more difficult problems including the recognition of familiar objects and procedures. For the case of relatively easier tasks (blue area) this basic influence of fact retrieval processes is complemented by additional variable fact retrieval processes, resulting in a more or less distinct processing pathway for problems that can be prominently solved by arithmetic fact retrieval (see above). With increasing task difficulty the influence of these variable, fact retrieval processes decreases, while the influence of magnitude related processes (red area) increases complimentarily. However, the basic fact retrieval processes are also assumed to take effect in difficult arithmetic. This is corroborated by results suggesting that participants break down complex problems into more tractable pieces (e.g., 42+53 in 2+3 = 5; [21]), which, in turn, may be retrieved from long-term memory. Moreover, amongst others, the fact-retrieval network included the RC, the hippocampus, the medial frontal cortex and the left angular gyrus. Parts of these structures have been reported to be affected in patients with a left-hemispheric lesion in the territory of the middle cerebral artery (MCA; for a review see [67]). Importantly, the circumscribed fact retrieval deficits often exhibited by patients with such a left-hemispheric MCA lesion can be accounted for by our model assuming an interruption of the pathways underlying arithmetic fact retrieval. The fact that these patients also experience severe problems in more difficult arithmetic can again be explained by our model. As basic arithmetic fact retrieval is involved in more difficult problems as well, a lack of arithmetic fact knowledge should influence difficult arithmetic as well.

In sum, the model presented in Figure 5 may explain the way *how* these two networks interact. Nevertheless, the question remains *through which anatomical structures* this interaction manifests itself. Although we were not able to tackle this question directly, there are several reasons to assume that the two networks are most likely connected at least by a link between the IPS and the AG. This proposition makes sense from a theoretical point of view and is also in line with recent data. As we described above, the IPS is assumed to be activated whenever we see, hear or operate with numbers [8], [68], [69]. This means, the IPS is generally involved in numerical tasks. This is also reflected in Figure 5. However, the IPS and the AG are not only located close to each other anatomically (in fact the IPS delineates the *border* of the upper part of the AG), but are also closely connected via association fibers (e.g., [37], [64] for tracking data). Moreover, there is a rich body of literature suggesting that the upper part of the anterior AG (PGa) is central for mental arithmetic [70], [71] and, especially, for differentiating familiar from untrained arithmetic problems [72].Taken together, due to various pieces of evidence from the literature, it is highly likely that the two networks are connected via links between the AG and the IPS.

Thus, our model provides a first theoretical sketch of how to integrate the anatomically distinct pathways of arithmetic fact retrieval and magnitude manipulations with the fact that these networks need to work closely together, because they were identified within the same task. In summary, we suggest that it may not be an either or question between arithmetic fact retrieval and magnitude manipulations, but rather a still unresolved question of how both networks operate together and how their interplay is adapted to task difficulty. Future studies are needed to investigate these questions.

### The retro-splenial cortex in arithmetic fact knowledge

Generally, the connectivity of the retro-splenial cortex (RC) with the AG and the medial frontal cortex seems to resemble the connectivity pattern of the so-called “default-mode network”, which is supposed to show higher activity during resting states than during a cognitive task (e.g., [37]). However, in the literature there is also evidence that RC responses (e.g., during tests of spatial navigation with memorized cues as well as autobiographical memory retrieval) are significantly above resting-state baseline, reflecting a valid engagement of the RC in performing such tasks (e.g., [73]–[75]). Furthermore and probably most important, the activation of the hippocampus in our fMRI data suggests that participants actively retrieved facts from long-term memory in the easier tasks. Thereby, these data argue against the default-mode network hypothesis, making the interpretation unlikely that the connectivity between areas involved in "easy arithmetic" merely reflects a higher involvement of the default-mode network due to reduced cognitive demands. Therefore, we wish to elaborate on the role of the RC in arithmetic fact retrieval in more detail.

Interestingly, the RC has not yet been specified anatomically for the TCM [8]. Nevertheless, we found it active in tasks with easy (verbally mediated) arithmetic problems. The main connections of the RC include links with the hippocampal formation, the parahippocampal region, and the parietal cortex (for a review see [61]). Thereby, the connections from the RC directly point to a role in memory, possibly subserving retrieval in declarative long-term memory. Furthermore, connections from RC include pathways to the prefrontal cortex, which provide an indirect route for hippocampal influences on the dorsolateral prefrontal cortex [76] as well as tracts to the medial prefrontal gyrus [77]. In the current study, all these areas were found to be involved in easy (verbally mediated) arithmetic problems. These findings point to a possible role of the RC in mental arithmetic, which seems to be a key member of a core network [61]. Among others, its functions have been suggested to include the recognition of familiarity, e.g., by detecting familiar voices and faces [78], episodic memory [73], and processing of objects strongly associated with a specific context [79]. Furthermore, functional studies of the RC of rodents consistently pointed to a role in learning using familiar cues [61].

Alongside these observations, we suggest that in easier addition problems, which may be solved more prominently by arithmetic fact retrieval, the task and/or the correct solution probe may be particularly familiar to participants (e.g., in 3+4 = 7 or 9 the ‘7’ may immediately catch the eye). The known connections of the RC with the hippocampal formation may further reflect that participants retrieve the solution to the problem (e.g., in 3+4 = ?) or to parts of the problem (e.g., the 3+4 from 13+24) from long-term memory. Also, the respective components of the task may appear more familiar and may be more strongly associated with the correct solution.

In summary, the retro-splenial cortex in mental arithmetic may point to retrieval from long-term memory for familiar (easy) addition problems. However, we wish to indicate that this may only be a first step towards a more comprehensive understanding of the involvement of the RC together with the medial frontal gyrus and the hippocampus in mental arithmetic. Functionally, this triangular network would make sense to be involved in the retrieval of arithmetic facts: the hippocampus is known to be involved in retrieval from long-term memory [80], while the retro-splenial cortex and the medial frontal gyrus have been repeatedly associated with the recognition of familiar objects or procedures (for a review see [61]. Considering evidence from fields other than numerical cognition such as research on autobiographical memory and episodic memory (e.g. [73]), we suggest that the RC and hippocampal structures may be a meaningful complementation of the current version of the TCM [4], [8].

### Representational characteristics of arithmetic fact knowledge

There is an ongoing debate about the representational code of arithmetic fact knowledge, While some authors claimed that arithmetic memory may be based on abstract or language independent representations [81]–[84] the dominant view in numerical cognition is that arithmetic facts (e.g., 2+3 = 5) are based on language-specific codes [8], [10], [85], [86], The present study is informative on this aspect as well. Not only did we find activation in language areas along the perisylvian sulcus for the case of easier, more retrieval related problems. Also the tracking results, indicating a prominently ventral pathway system, which proved to be largely identical to the system recently identified for language processing [48], [49], clearly suggest that the retrieval of arithmetic facts may be verbally mediated.

### Interpretation of the data in terms of the dual loop model

Functionally, the observed dorsal and ventral tracts relate nicely to the so-called “dual loop system”, which has been proposed for the case of language processing [49]: according to the dual loop system, the dorsal system is supposed to rather subserve processing of sequences of mental objects/items, whereas the ventral system is assumed to be dedicated to categorical decisions dependent on item structure. In particular, the dorsal stream has been associated with a more general (time dependent) capacity to analyse a sequence of segments, either in time or in space, as well as fast on-line integration between sensory event information and ‘‘internal models or emulators’’ independent of modality [49]. This notion may be transferred to the case of mental arithmetic. For instance, whenever a mental addition problem requires updating the place-value structure of an Arabic digit, the sequence of the segments of the Arabic multi-digit number has to be evaluated and modified accordingly, probably using the SLF II. In case the addition problem gets even more difficult (e.g., with increasing size of the operands), the SLF III may be additionally recruited connecting the intraparietal cortex with BA 44, which also subserves working memory [48]. On the other hand, the ventral route has been suggested to be involved in the identification of structural relations independent of the modality and of the time or sequence of occurrence of the elements [49]. Thus, the ventral route in difficult mental arithmetic may not only be devoted to the application of rules but also, more generally, to the categorical identification of the digits involved as specific integers, irrespective of the sequence of these integers. In summary, general principles associated with dorsal and ventral processing pathways, as identified in studies on language processing [48], [49], may be adapted to the case of mental arithmetic. Thereby, our data point to more basic underlying computational mechanisms carried out in the dual loop system, which may be instrumental in various domains.

### Limitations and perspectives

Even though this is the first study to investigate the neural connections within the fronto-parietal network for numerical cognition, there are some points to be kept in mind. First, it has to be noted that the fMRI and the DTI samples are not fully comparable with respect to demographic variables such as gender. In the literature, differential effects have been reported on numerical processing for male and female participant groups. However, the vast majority of evidence concerns effects of lateralization (e.g., [87]). Since the main results of the study stem from an intrahemispheric tracking (from the left hemisphere), we are confident that the gender difference between the fMRI (only males) and the DTI sample (females and males) does not affect the main message of the manuscript substantially. Additionally, it should be noted that recent neuroimaging studies seem to indicate that functional cerebral asymmetries due to the menstrual cycle of women may be even more pronounced than differences in lateralization between the sexes (e.g., [88], [89]).Nevertheless, we suggest that this point should be addressed in future studies to evaluate the generalizability of the observed networks for both sexes.

Second, it is important to emphasize that probabilistic fiber tracking can only provide probability measures for deciding on whether specific anatomical tracts may connect two regions. It needs to be noted that the lack of a connection between two seed regions in the analysis does not exclude that there is nevertheless a connection. Principally, the method of probabilistic fiber tracking is suited to complement the anatomical specification of functionally pre-defined networks. Therefore, the plausibility of the identified networks needs to be evaluated theoretically and cannot be inferred from the method itself. At least for the network of arithmetic fact retrieval the plausibility of the identified tracts seems to be high. For arithmetic fact retrieval we used seed regions, which are identical (+/- some few mm) to the coordinates reported for language studies (e.g., [49]). Therefore, the connections found in the present study for fact retrieval seem plausible as they largely matched connections in language studies. When seed A and B are incorporated in both, a tracking study on arithmetic facts but also in a study on language processing, it is obvious that the observed fiber tracking results for arithmetic imply the processing of verbal information along the identified tracts, as indicated by the identical tracking results in language studies [48], [49]. Additionally, this is also plausible from a theoretical point of view. According to the TCM, arithmetic fact knowledge is assumed to be represented in a verbal code and the functional network subserving arithmetic fact retrieval is assumed to rely on language areas [7]. While this argues for the plausibility of the obtained fiber connections for the case of arithmetic fact retrieval, the plausibility for the magnitude-related network is less clear and can only be derived indirectly because there is no fiber tracking study investigating magnitude related processing so far. There is only evidence for single connections from separate studies. Nevertheless, these evidences are in line with our data: for instance, in the study by Krueger et al. [36] similar connectivity was substantiated for number processing in the IPS with the SMA. Also from the study by Caspers et al. [64], both dorsal and ventral connections between the intraparietal cortex and frontal areas such as BA 44 and BA 45 were detected by combining DTI with cytoarchitectonical evidence as well as receptor fingerprints. Finally, the overall network obtained for number magnitude processing is in line with the propositions of the TCM. Third, the issue of interdependence between the two networks is a highly interesting question, which may be addressed in future studies using different approaches such as deterministic fibertracking combined with analyses of the whole brain connectivity pattern. However, future studies are needed to further elaborate on these issues.

### Conclusions

The currently most influential model in numerical cognition (TCM, [4], [8] indicates numerical cognition to be a case of multi-modular and distributed processing. However, so far there has been no attempt to (i) specify the anatomo-functional connection between the different cortex areas assumed to underlie numerical cognition, nor (ii) to integrate the suggested fronto-parietal network of numerical cognition into established frameworks of cognitive information processing such as the one differentiating dorsal and ventral processing pathways.

The current study addressed these issues by means of probabilistic fiber tracking. We used seed regions motivated both theoretically (TCM, [4], [8]) as well as empirically (re-analysis of a recent fMRI study on mental addition [34]. Particular interest was paid to a differentiation of neural correlates and connectivity for more difficult and relatively easier addition problems and their theoretically associated reliance on either magnitude manipulations or verbally mediated fact retrieval, respectively–with magnitude manipulations involving dorsal pathways and arithmetic fact retrieval involving ventral pathways.

In line with these hypotheses, the present data suggest that magnitude- and fact retrieval-related processing are indeed subserved by two largely separate networks, which differ not only in localization of activation but also in the connections between them. However, although both networks involved dorsal and ventral pathways, these pathways actually differed to a large degree. Nevertheless, it is important to note that, even though seemingly distinct anatomically, these networks seem to operate as a functionally integrated circuit for mental calculation. This can be concluded because the networks were identified applying parametric analyses to the same task.

From this we conclude that general principles associated with dorsal and ventral processing paths, as identified in studies on visual processing [40], [42], the attentional system [50] as well as language processing [48], may be adapted to the case of mental arithmetic. Thereby, our data point to more basic underlying computational mechanisms in a dual loop system, which may be instrumental for various domains [49].
